# Cosmetic Contact Sensitivity in Patients with Melasma: Results of a Pilot Study

**DOI:** 10.1155/2014/316219

**Published:** 2014-07-14

**Authors:** Neel Prabha, Vikram K. Mahajan, Karaninder S. Mehta, Pushpinder S. Chauhan, Mrinal Gupta

**Affiliations:** Department of Dermatology, Venereology & Leprosy, Dr. R. P. Govt. Medical College, Kangra, Tanda, Himachal Pradesh 176001, India

## Abstract

*Background*. Some of the patients with melasma perhaps have pigmented cosmetic dermatitis. However, cosmetic contact sensitivity in melasma remains poorly studied particularly in the Indian context.* Objectives*. To study cosmetic contact sensitivity in patients with melasma. *Materials and Methods*. 67 (F : M = 55 : 12) consecutive patients with melasma between 19 and 49 years of age were patch tested sequentially during January–December, 2012, with Indian Cosmetic and Fragrance Series, Indian Sunscreen Series, *p*-phenylenediamine, and patient's own cosmetic products. *Results*. 52 (78%) patients were in the age group of 20–40 years. The duration of melasma varied from 1 month to 20 years. Centrofacial, malar, and mandibular patterns were observed in 48 (72%), 18 (27%), and 1 (1%) patients, respectively. Indian Cosmetics and Fragrance Series elicited positive reactions in 29 (43.3%) patients. Cetrimide was the most common contact sensitizers eliciting positivity in 15 (52%) patients, followed by gallate mix in 9 (31%) patients and thiomersal in 7 (24%) patients. Only 2 of the 42 patients showed positive reaction from their own cosmetics while the other 5 patients had irritant reaction. Indian Sunscreen Series did not elicit any positive reaction. *Conclusion*. Cosmetics contact sensitivity appears as an important cause of melasma not associated with pregnancy, lactation, or hormone therapy.

## 1. Introduction

The use of cosmetics and skin care products for grooming of both men and women has seen tremendous rise the world over in the last few years. Fairness creams/lotions and sunscreen are perhaps the most sought after cosmetics for daily use particularly in India and other Asian countries. The cosmetics are different from drugs, they lack diagnostic and therapeutic properties, and they are used topically to cleanse, beautify, perfume, protect from body odors, or promote attractiveness. Additionally, the cosmetic allergens may come in contact with skin from a product used by the partner/other persons, airborne vapors/droplets, or accidental transfer by hands to more sensitive areas like eyelids and after contact with an allergen-contaminated surface. Occasionally, patients may experience numerous allergic reactions to cosmetics or photosensitivity from photo-allergens in a cosmetic product and exposure to sunlight especially ultraviolet (UV)-A. The reported prevalence of cosmetic allergy varied between 29 and 36% during 1999 to 2008 while fragrances and preservatives were the most common allergens [1–4]. Similarly, sunscreen chemicals, used as such or as ingredients in other cosmetics, are a common cause of irritant or allergic contact dermatitis. They often interact with* Myroxylon pereirae* (balsam of Peru) and/or fragrance additives (cinnamic acid, cinnamic aldehyde, and cinnamon oils) and elicit contact reactions [5, 6]. Moreover, benzophenones are well-known cause of photoallergic reactions [6]. However, cosmetics have been rarely implicated to cause melasma [7]. Pigmented cosmetic dermatitis, as proposed by Nakayama et al. [8], is a variant of pigmented contact dermatitis where cosmetic ingredients are the primary allergens and the face is involved predominantly. Clinically, diffuse or patchy brown hyperpigmentation occurs over cheeks and/or forehead or the entire face making its differentiation difficult from melasma. However, this aspect of cosmetic contact sensitivity in melasma remains poorly studied. In this pilot study, we present our observations on cosmetic contact sensitivity in patients with melasma.

## 2. Material and Methods

67 (F : M = 55 : 12) patients aged ≥18 years with melasma were enrolled for the study during January–December 2012 after a written/informed consent. The study was approved by the Institutional Protocol Review Board and Institutional Ethics Committee (Registration no. ECR/490/Inst/HP/2013). Pregnant or lactating women and patients taking oral contraceptives/other medications or having other pigmentary disorders, endocrinopathies, or family history of melasma were excluded. Details about age, sex, occupation, onset, duration, and progress of melasma, clinical patterns of melasma, aggravating factor, use of cosmetics, and medications were recorded. All patients were patch tested sequentially by Finn chamber method using Indian Cosmetic and Fragrance Series (Table 1) and IndianSunscreen Series (Table 2).

Additionally, personal cosmetic products brought by the patients were also patch tested (as is) along with* p*-phenylenediamine (PPD, 1.0% pet), a constituent of commonly used hair coloring agents. The patch tests were applied on upper back and the patients returned for reading of results after 48 hrs (D2) and 72 hrs (D3). The results were graded according to the International Contact Dermatitis Research Group criteria [9]. Reactions persisting on D3 were considered positive for final analysis. Other 10 volunteers were also patch tested similarly as controls. They were using similar cosmetics and did not have melasma. Relevance of positive patch test results was determined clinically. Side effects (adhesive tape reaction, discomfort and itching, flare up of dermatitis, angry back phenomenon, active sensitization, and pigment alteration at test site), if any, were noted.

## 3. Results and Observations

The study comprised 55 (82%) females aged between 19 and 49 years and 12 (17.9%) males in the age group of 20 to 32 years. 52 (77.6%) patients were in the age group of 20–40 years and constituted the majority. The duration of melasma varied from 1 month to 20 (mean 3.3 years) years. The majority, 38 (56.7%) patients, had melasma for 1 to 5 years and 19 (28.3%) patients had melasma for <1 year while its duration was more than 5 years in 10 (14.9%) patients, respectively. All patients had well delineated clinical patterns of melasma; 48 (71.6%) patients had centrofacial pattern, 18 (26.8%) patients had malar pattern, and one (1.4%) patient had mandibular pattern (Table 3).

**Figure 1:**
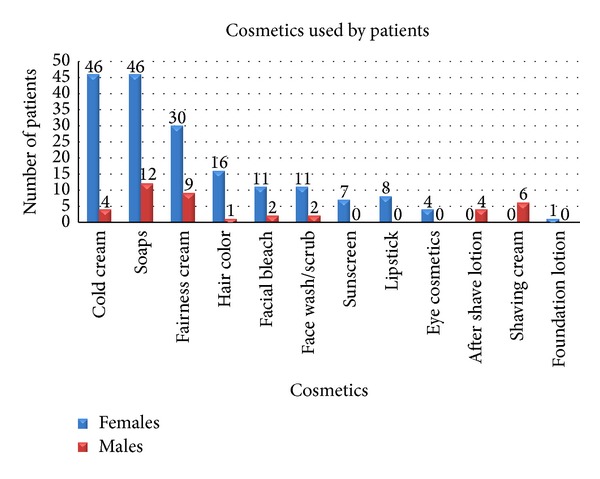
Common cosmetics used by the patients.

Common cosmetics/skin care products used were cold creams and skin moisturizers (50 patients), medicated soaps (58 patients), fairness creams (39 patients), hair colors (17 patients), facial bleach (13 patients), and sunscreens (7 patients), respectively (Figure 1).

All 67 patients were patch tested with Indian Cosmetics and Fragrance Series but only 46 patients turned up for sequential patch testing with Indian Sunscreen Series. Patch test results with Indian Cosmetics and Fragrance Series were positive in 29 (43.3%, *n* = 67) patients and none showed positive result from Indian Sunscreen Series. Cetrimide was the most common contact sensitizers in 15 (52%, *n* = 29) patients followed by gallate mix in 9 (31%) patients, respectively. Thiomersal elicited positive results in 7 (24%) patients among 39 patients using fairness creams (Fair & Lovely, Fair & Handsome, Garnier Lite, Olay). Isopropyl myristate, jasmine synthetic, sorbic acid, bronopol, chloroacetamide, vanillin, 2-(2-hydroxy-5-methyl-phenyl) benzotriazole, germall 115, quaternium 15, triethanolamine, geranium oil, butylated hydroxyanisole, and hexamine elicited positive reactions in one patient each. Polysenstivity, that is, positive patch test reactions to ≥2 allergens, was observed in 11 (38%, *n* = 29) patients; 5 patients had sensitivity to 2 allergens, 5 patients to 3 allergens, and 1 patient to 5 allergens, respectively. One patient showed sensitivity to gallate mix, thiomersal, and jasmine synthetic simultaneously. One patient showed sensitivity to gallate mix, thiomersal, and cetrimide simultaneously. One patient showed sensitivity to chloroacetamide, phenyl salicylate, and cetrimide simultaneously. One patient showed sensitivity to bronopol, cetrimide, and foundation lotion. One patient showed sensitivity to PPD and gallate mix. Two patients each were positive to gallate mix and cetrimide simultaneously.

42 patients were patch tested with their own cosmetics/skin care products “as is.” One male patient showed positive reaction to fairness creams (“Fair & Handsome” and “Fair & Lovely” cream). One female patient showed positive reaction to her foundation lotion. Five patients showed irritant reaction to “Lifebuoy” soap and one each to a soap containing sandalwood oil (Santoor soap) and fairness creams (as above), face wash (Fair & Lovely and Soundarya face wash), and after shave lotion (Gillete), respectively.

Ten (M : F 5 : 5) controls aged between 26 and 48 years were healthy volunteers or attendants accompanying the patients. One controls subject had positive reactions from gallate mix, polyoxyethylene sorbitan, sorbitan sesquioleate, and stearyl alcohol. PPD elicited positive reaction in another female who never had contact dermatitis clinically despite using hair colors.

## 4. Discussion

Melasma is a common acquired hypermelanosis involving the face, and being of long-standing nature has significant effect on psychology and quality of life. Although the exact prevalence of melasma is unknown, it accounts for 0.25 to 4% of the patients seen in dermatology clinics in South East Asia and is also a common pigment disorder among Indians [10, 11]. The disease affects all races but Hispanics and Asians predominate [12]. Genetic predisposition, pregnancy, oral contraceptives, endocrine dysfunction, hormone treatments, or exposure to UV light is the most implicated etiologic factors in melasma [7]. Drugs containing phototoxic agents, phenothiazines, and anticonvulsants have been particularly linked to melasma. However, cosmetics have been rarely considered in the list of causes of melasma [7, 10]. There is a predilection for the involvement of cheeks, forehead, upper lip, nose, chin, and sometimes neck as well. However, three distinctly recognized clinical patterns include centrofacial, malar, and mandibular. The most common centrofacial pattern was seen in 55% and 75% while malar pattern and the mandibular pattern occurred in 43**%** and 24% and 2**%** and 1.5% patients in two separate studies, respectively [12, 13]. Melasma affects predominantly women, men comprising only 10% of all cases or perhaps men consult less often for aesthetic motives, but it rarely manifests before puberty [12, 14]. The majority 52 (77.6%) patients in our study were in the age group of 20–40 years and predominately comprised females (82%) in the age group of 19–49 years corroborating above clinicoepidemiological findings. Similarly, they also did not differ in duration and age of onset from what has been reported previously [12]. Men comprised only 18% in our study as compared to 10% of all cases in a previous study [14]. Similarly, our patients also had centrofacial pattern in 48 (71.6%), malar pattern in 18 (26.8%), and mandibular pattern in 1 (1.4%) patients in order of frequency corroborating with earlier studies [12, 13].

**Figure 2:**
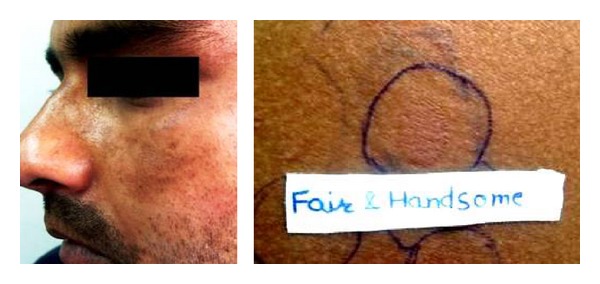
Irritant patch test (Janus type) reaction from Fair & Handsome cream in a male with malar pattern.

**Figure 3:**
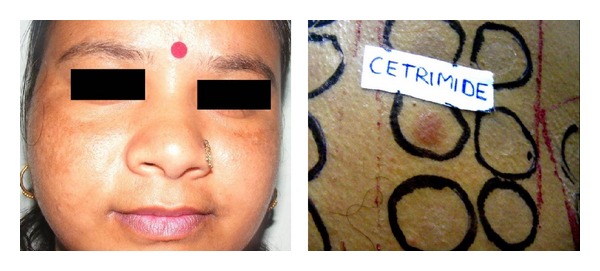
A patient with malar pattern of melasma and positive patch test from cetrimide.

**Figure 4:**
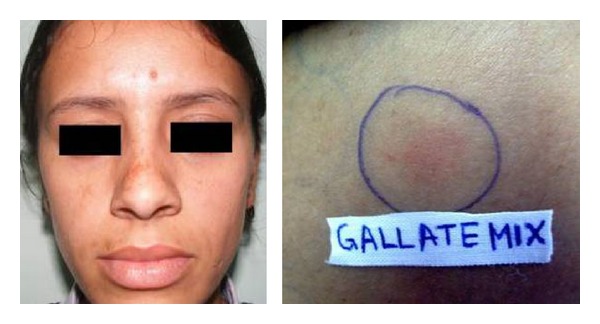
A patient with malar pattern of melasma and positive reaction from gallate mix.

Cosmetics are complex mixtures of perfumes, preservatives, emulsifiers and stabilizers, various lipids, and higher alcohols. Various chemicals in cosmetics (colophony, PPD, balsam peru, cetostearyl alcohol, lanolin, bees wax, formaldehyde, fragrances, musk mix, vanillin, rose oil, triclosan, or other antiseptics) have been implicated to cause primary irritant reactions, allergic contact dermatitis, photoallergic contact dermatitis, contact urticaria, pigment alteration, photosensitivity, brittle hair and nails, and so on (Figure 2). Hyperpigmentation, as in Berloque dermatitis, Riehl's melanosis, poikiloderma of Civatte, and erythrosis pigmentata faciei of Brocq, has been attributed to bergamot oil in eau-de-Cologne or from tars in cosmetics. Our 29 (43.2%) patients with melasma showed positive patch test results from cosmetic chemicals. Cetrimide was the most frequent allergen accounting for 52% of the positive results (Figure 3). Cetrimide is an antiseptic and major formulation excipient chemical in cosmetics and reported to elicit positive reactions in 12% of 50 patients with cosmetic dermatitis [15]. The formulation excipients are inert substances that serve to solubilize, emulsify, sequester, thicken, foam, lubricate, or color the active component in a product. However, they can be responsible for allergic contact dermatitis or can act as irritants when used in higher concentrations particularly in locations of direct contact with the allergen-containing products. Our patients who were patch test positive with cetrimide were using various facial cosmetics (cold creams, fairness creams, antiseptic soaps, face wash/scrubs, shaving creams, and aftershave lotions). One male patient with cetrimide positivity had also reported irritant reaction to aftershave lotion. However, we tend to agree with Beltrani et al. [16] that predicting the precise allergen in suspected cosmetics is difficult in view of ubiquity of these chemicals in cosmetic products. Dodecyl gallate, octyl gallate, and propyl gallate (gallate mix) are antioxidant substances used as preservatives in cosmetics (lipstick, liposome containing creams, body lotions, facial moisturizers, facial cleanser, body wash and cleansers, hair conditioners, and foundation lotions), foods, and the topical pharmaceutical preparations. The use of liposome containing creams has been implicated for rise in propyl gallate allergy observed in patients patch tested from 1988 to 2005 over the previous decade [17, 18]. Gallate mix was the second most frequent allergen eliciting positive results in 31% of our patients who have been using various facial cosmetics/skin care products (Figure 4).

**Figure 5:**
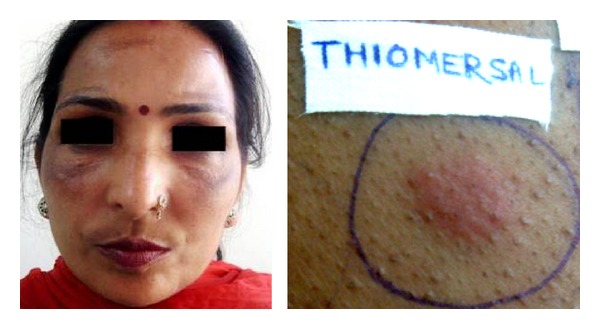
A patient with centrofacial melasma and positive reaction from thiomersal.

Skin lightening soaps and fairness creams usually contain inorganic mercury (ammoniated mercury) while organic mercury compounds (ethyl mercury or thiomersal, phenyl mercuric salts) are used as preservatives in cosmetics, eye drops, contact lens solutions, vaccines, and antiseptics. Thiomersal is considered uncommon allergen and the reported thiomersal contact sensitivity in patients of cosmetic dermatoses or pigmented cosmetic dermatitis varies from 8% to 77% [19–21]. However, discretion is recommended in interpretation of positive patch test reaction to thiomersal as primary sensitization may be from childhood vaccination. Nevertheless,chronic use of topical mercury may itself cause increased pigmentation due to accumulation of mercury granules in the dermis via absorption through hair follicles and sebaceous glands. Boonchai et al. [1] also observed that ammoniated mercury showed a significantly increased tendency to cause cosmetic allergies over a 10-year period. Interestingly, mercury is rarely listed as a component of commercially available cosmetics. Al-Saleh and Al-Doush [22] after analyzing 38 commercially available skin lightening creams in 1997 noted that 45% of the tested samples contained mercury at levels far surpassing 1 ppm (the maximum permitted limit by FDA). More recently in 2005, they also analyzed “Fair & Lovely” fairness cream and found traces of mercury that was otherwise not its listed component [23]. Thiomersal was third common allergen in order of frequency eliciting positive reactions in our 7 (24%) patients who were using various fairness creams (Figure 5).

**Figure 6:**
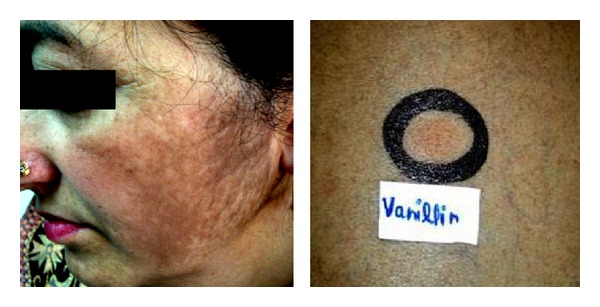
Positive patch test from vanillin in a patient with diffuse-to-reticulated mandibular pattern of melasma.

Another patient who had positive patch test from phenyl salicylate, chloroacetamide, and cetrimide was using 5 different varieties of face creams. Phenyl salicylate is a preservative, denaturant for alcohol and fragrance ingredient in cosmetics, face and hand creams, mouthwashes, and sunscreen preparations. It has a pleasant odor somewhat similar to that of oil of wintergreen. There are reports of cheilitis from lip salve containing phenyl salicylate wherein both lip salve and phenyl salicylate elicited positive patch test reactions [24, 25]. Similarly, Fimiani et al. [26] reported a 17-year-old woman of hand dermatitis from galenic cream and showed positive reactions to both phenyl salicylate and her galenic cream but not to the petrolatum. However, our patient had no positivity from her cosmetic creams. PPD is a strong sensitizer and sensitization may occur from PPD in textile or fur dyes, black rubber, temporary tattoos, photocopying, and printing inks. In addition to PPD induced acute allergic dermatitis, uncommon presentations such as pigmentary changes too have been ascribed to its use. Dandale et al. [21] documented positive reactions from PPD in 8.6% patients of facial melanosis. Mehta et al. [27] also described a case of pigmented contact cheilitis from PPD. Our 2 patients and one control had positive reaction to PPD but never had clinical contact dermatitis despite using hair colors in the past or perhaps being subtle clinically it remained unnoticed. Jasmine synthetic, chloroacetamide, isopropyl myristate, vanillin, bronopol, sorbic acid, 2-(2-hydroxy-5-methyl-phenyl) benzotriazole, germall 115, hexamine, quaternium 15, geranium oil, butylated hydroxyanisole, and triethanolamine, the common additives to cosmetics/skin care products, appear to be uncommon sensitizers. One female patient who had positive patch test from jasmine synthetic, a common fragrance in cosmetics, also showed positive reaction to gallate mix and thiomersal. She was using “Ayur” body lotion and “Fair & Lovely” fairness cream but had no positive reaction from them. Positive reactions from gallate mix and thiomersal in her could be from reasons vide supra. Chloroacetamide, a common preservative, is a well known cause of cosmetic allergy from baby lotion, cleansing lotion, eye cream, massage cream, facial cream, hand lotion, and antiwrinkle serum in Europe [28]. Although in our study none of the patient's own cosmetics elicited positive reaction, it is possible that our patient was sensitized from other cosmetics or pharmaceuticals used in the past. The reported sensitivity from isopropyl myristate, an emollient, fragrance, and skin-conditioning agent, was 1.2% in 244 patients with cosmetic contact dermatitis in a study from Israel [29]. Although our patient showed positive patch test reaction from isopropyl myristate, cosmetic cream itself did not elicit positive reaction in her despite isopropyl myristate being one of the listed ingredients in “Fair & Lovely” cream that she had been using for over 6 years. Perhaps this ingredient is present in much lower concentration in finished cosmetics product than used for patch testing. Although vanillin, a substituted aromatic aldehyde and a fragrance, is known to induce skin sensitization in humans [30], it is often considered secondary allergen in patients sensitized to* Myroxylon pereirae* and positive reactions to vanillin (pure or 10%) were reported in 8/142 and 21/164 of such patients [31]. Vanillin elicited positive reaction in a female (Figure 6) who had been using various cosmetics (cold creams, soap).

**Figure 7:**
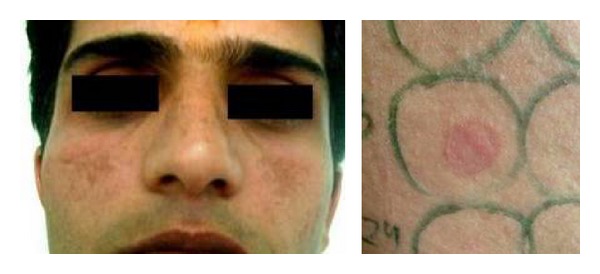
Positive patch test from sorbic acid in a patient with malar pattern of melasma.

Bronopol, a formaldehyde-releasing preservative in topical medications and cosmetics specially the foundation lotion, had elicited 10 (0.12%) irritant and 38 (0.47%) allergic reactions in a series of 8149 patients who were patch tested in seven European contact dermatitis clinics; only 17 (0.21%) were considered to be of current or past clinical relevance [32]. Bronopol caused contact sensitization in one of 202 patients with cosmetic dermatitis in another series [33]. The only patients who had positive reaction to bronopol in our series also had positive reaction from her foundation lotion. Sorbic acid, a common preservative in antiaging cream, cleansers, shampoos, exfoliant/scrub, shaving creams, and aftershave lotion, elicited positive reaction in one male patient who showed positive reaction to sorbic acid (Figure 7) and was using face wash (Johnson's), walnut scrub (Everyouth), after shave, and shaving cream (Gillette). Silva et al. [34] patch tested 147 patients with suspected cosmetic dermatitis and sorbic acid produced positive reactions in 9 patients.

Another female patient who was using fairness (Fair & Lovely) cream, various soaps, and shampoo had positive reaction from cetrimide, 2-(2-hydroxy-5-methyl-phenyl) benzotriazole, germall 115, hexamine, and quaternium 15. She also showed irritant reaction to “Fair & Lovely” fairness cream and “Lifebouy soap.” 2-(2-Hydroxy-5-methyl-phenyl) benzotriazole is used as a UV absorber in cosmetics and dental materials and has caused contact sensitivity in 1 patient in an earlier study of 50 patients with cosmetic dermatitis [15]. Germall 115 (imidazolidinyl urea) is a formaldehyde-releasing preservative in creams/lotions, hair conditioner/shampoos, and deodorants while hexamine is used as a solvent in cosmetics. Quaternium 15, a preservative in creams/lotions, shampoos, and soaps, elicited positive reaction in 1 (2.8%) of 35 patients of cosmetic dermatoses [20]. Geranium oil, another fragrance ingredient, caused positive reaction in our one male patient along with positive reactions from gallate mix and phenyl salicylate and he was using face wash, shaving cream, after shave lotion, soaps and hair color. Geranium oil contact sensitivity has also been reported previously in 10% of 50 patients with cosmetic dermatitis [15]. One male patient who had positive patch test from butylated hydroxyanisole (antioxidant in cosmetics), triethanolamine (surface-active agent in soaps, shampoos), and gallate mix was using fairness creams, antiacne (herbal) cream, and soaps but elicited no positive reaction from these products. The reported positivity from these cosmetic ingredients is 8.7% in patients with cosmetic contact dermatitis [35].

Sunscreens are common causes of photoallergic contact dermatitis and are frequently present in cosmetics such as moisturizers, lip and hair preparations, and foundations. They are capable of causing allergic contact dermatitis even in the absence of photo activation [16]. None of our 46 patients, however, showed positive results from sunscreen series. As photo patch testing was not performed, whether melasma among them is from photo allergic contact dermatitis remains unknown.

Overall, it was observed that there was dissociation between the patch test results from individual cosmetics ingredients and the cosmetic product when patch tested as such in our 42 patients. Dogra et al. [36] also made similar observations that ingredients of cosmetics showed more frequent sensitivity as compared to the cosmetics applied as such perhaps because of exposure to similar ingredients present in other products/medicaments and presence of ingredients in much lower concentration in finished products/cosmetics. Moreover, manufacturers usually do not list most of the ingredients on the package. This is quite evident in a study of allergic contact dermatitis from gallates and a skin repair cream was one of products suspected of causing allergic reactions [37]. The list of ingredients of current packaging did not specify presence of gallates whereas previous older packaging stated that it contained propyl gallate. The researchers could not ascertain whether the formulation had changed or the product contained such miniscule quantities that its name was deleted from the ingredient list. Interestingly, none of our patients experienced symptoms of contact sensitivity from their cosmetics or attributed melasma to use of cosmetics. It has been suggested that there are perhaps subtle signs of preceding dermatitis in few patients and others may not observe any skin changes or itching attributable to the cosmetic use prior to or during the development of the pigmentation [38].

## 5. Conclusion

Pigmented cosmetic dermatitis and cosmetics contact sensitivity should be considered in the etiologic factors when melasma is not associated with pregnancy, lactation, or hormone therapy. However, some of these cases having diffuse-to-reticulated pattern of hyperpigmentation (brown, slate-gray, gray-brown, red-brown, or blue-brown depending upon the causal agent) and diagnosed clinically as melasma are perhaps due to pigmented cosmetic dermatitis. It is also possible that positive patch test results to various cosmetic or their ingredients, listed or unlisted, are coincidental or false positive but the hyperpigmentation is primarily postinflammatory as has been suggested by Nakayama et al. [8]. Sun exposure only deepens the pigmentation further. Accordingly, the cosmetics perhaps cause low-grade inflammation and hyperpigmentation by way of cytolysis and melanin incontinence at basal layer level following irritant reaction or after absorption of allergen from daily application in concentrations enough to elicit contact hypersensitivity. This is also evident in our 2 patients and one control having positive reaction to PPD without apparent clinical contact dermatitis despite using hair colors previously. As manufacturers do not list most of the ingredients in a cosmetic product, the relevance of positive reactions may not possibly be ascertained in all. Furthermore, dissociation between the patch test results from individual cosmetics ingredients and the cosmetic product when patch tested as such could be due to presence of ingredients in much lower concentration in finished products of cosmetics [37]. Avoidance of cosmetic contact hypersensitivity is perhaps a first step in preventing/treating melasma.

## Figures and Tables

**Table 1:** Indian Cosmetic and Fragrance Series∗.

Sr. number	Allergen
1	Abitol (10%)
2	Amerchol L 101 (50%)
3	Benzyl alcohol (10%)
4	Benzyl salicylate (10%)
5	Bronopol (0.25%)
6	Butylated hydroxyanisole (BHA) (2.0%)
7	Butylated hydroxytoluene (2.0%)
8	Cetyl alcohol (5.0%)
9	Chloroacetamide (0.2%)
10	Chloroxylenol (0.5%)
11	Gallate mix (1.5%)
12	Geranium oil (2%)
13	Benzophenone (10%)
14	Drometrizole (1.0%)
15	Imidazolidinyl urea (2.0%)
16	Isopropyl myristate (2.0%)
17	Jasmine absolute Egyptian (2.0%)
18	Lavender absolute (2.0%)
19	Musk mix (3.0%)
20	Phenyl salicylate (1.0%)
21	Polyoxyethylene sorbitan (5.0%)
22	Rose oil (2.0%)
23	Sorbic acid (2.0%)
24	Sorbitan monooleate (Span 80) (5.0%)
25	Sorbitan sesquioleate (arlacel 83) (20.0%)
26	Stearyl alcohol (30.0%)
27	Tert-butyl hydroquinone (1.0%)
28	Thiomersal (0.1%)
29	Triclosan (2.0%)
30	Triethanolamine (2.0%)
31	Vanillin (2.0%)
32	Oleamidopropyl dimethylamine (0.4%)
33	Cetrimide (0.5%)
34	Jasmine synthetic (2.0%)
35	Hexamine (2.0%)
36	Control (100%)
37	Chlorhexidine digluconate (0.5%)
38	Phenyl mercuric acetate (0.01%)
39	Cocamidopropyl betaine (1.0%)
40	Diazolidinyl urea (germall II) (2.0%)
41	Ethylene diamine dihydrochloride (1.0%)
42	Quaternium 15 (Dowiell 200) (1.0%)
43	Propylene glycol (5.0%)
44	Kathon CG (1.3%)

**Table 2:** Indian Sunscreen Series∗.

Sr. number	Allergen
1	4-Tert-butyl-4-methoxy-dibenzoyl-methane (10%)
2	Homosalate (5%)
3	PABA (10%)
4	3-(4-Methylbenzylidene) camphor (10%)
5	2-Ethylhexyl-4-dimethyl-aminobenzoate (10%)
6	Benzophenone-3 (10%)
7	2-Ethyl hexyl-4-methoxycinnamate (10%)
8	2-Hydroxy-4-methoxy-4-methyl-benzophenone (10%)
9	Phenyl benzimidazole sulfonic acid (10%)
10	Octyl triazone (10%)
11	Octyl triazone (10%)
12	Drometrizole trisiloxane (10%)
13	Octocrylene (10%)
14	Octyl Salicylate (5%)
15	Ethylhexyl triazone (10%)
16	Isoamyl-p-methoxy cinnamate (10%)
17	Bis-ethylhexyloxyphenol methoxyphenyl triazine (10%)
18	Methylene bis-benzotriazolyl tetramethyl butyl phenol (10%)
19	2-(4-Diethylamino-2 hydroxybenzoyl) benzoic acid hexylester (10%)
20	Diethyl hexyl butamido triazone (10%)

*Note: both Indian Cosmetic and Fragrance Series and Indian Sunscreen Series are recommended by Contact Dermatitis and Occupational Dermatoses Forum of India and were purchased from Systopic India Limited, New Delhi (India).

**Table 3:** Clinical patterns of Melasma as observed in this study.

Clinical patterns of Melasma	Definition	Number of Patients
(1) Centrofacial	Pigmentation on cheeks, forehead, upper lip, nose, and chin	48 (71.6%)
(2) Malar	Pigmentation present only on cheeks and nose	18 (26.8%)
(3) Mandibular	Pigmentation on ramus of the mandible	1 (1.4%)
